# A new species of *Cyanea* Gaud. (Lobelioideae, Campanulaceae) from Maui, Hawai`i

**DOI:** 10.3897/phytokeys.167.55107

**Published:** 2020-11-20

**Authors:** Hank Oppenheimer

**Affiliations:** 1 Plant Extinction Prevention Program, Pacific Cooperative Studies Unit, University of Hawai`i, PO Box 909, Makawao, HI 96768 USA University of Hawai`i Makawao United States of America

**Keywords:** conservation, Hawaiian Islands, IUCN Red List, Plant Extinction Prevention Program

## Abstract

*Cyaneaheluensis* H. Oppenheimer, **sp. nov**., a new, narrowly distributed endemic species, is herein described and illustrated with line drawings and digital field photos. It is currently known from a single mature plant and is restricted to the upper Helu planeze on leeward Mauna Kahalawai, West Maui, Hawaiian Islands. It differs from all other species of *Cyanea* Gaudich. with its narrow, shallowly lobed leaves, gently curved muricate corollas, and undulate sepals caducous in fruit. A key to the new species and its congeners on Mauna Kahalawai is provided. Its conservation status and efforts to propagate the species are discussed.

## Introduction

The Hawaiian lobeliods are the largest plant clade restricted to any archipelago, with *Cyanea* being the most species-rich genus within that clade. It is also the largest genus in Hawai`i, and originated from a single introduction of 8–10 Mya (Givnish et al. 2008). As currently circumscribed, the woody lobelioid genus *Cyanea* Gaudich. (including *Rollandia* Gaudich.) comprises 80 species (Oppenheimer and Lorence 2012; Spork-Koehler et al. 2015) all endemic to the Hawaiian Islands where they occur in wet and mesic forests. Most have a very narrow distribution, and are single-island endemics, or restricted to a single volcano. *Cyanea* was first described by Gaudichaud-Beaupré (1829) based on the type species *C.grimesiana* Gaudich. The genus was later treated in Rock’s (1919) monographic study of the Hawaiian Lobelioideae in which he recognized 52 species in 5 sections. Wimmer (1943) later recognized only 3 sections in his monograph of Campanulaceae. Lammers (1990) revised the Hawaiian members and also recognized 52 species, but stated relationships within *Cyanea* remained poorly understood and consequently did not recognize any formal sections. Givnish et al. (1995) recognized an orange fruited clade and a purple fruited clade. Recent exploration and collecting efforts in poorly explored, often rugged or remote regions in the Hawaiian Islands, continue to yield undescribed species of *Cyanea* (Lammers and Lorence 1993; Lammers 2004; Oppenheimer and Lorence 2012; Spork-Koehler et al. 2015).

**Figure 1.**
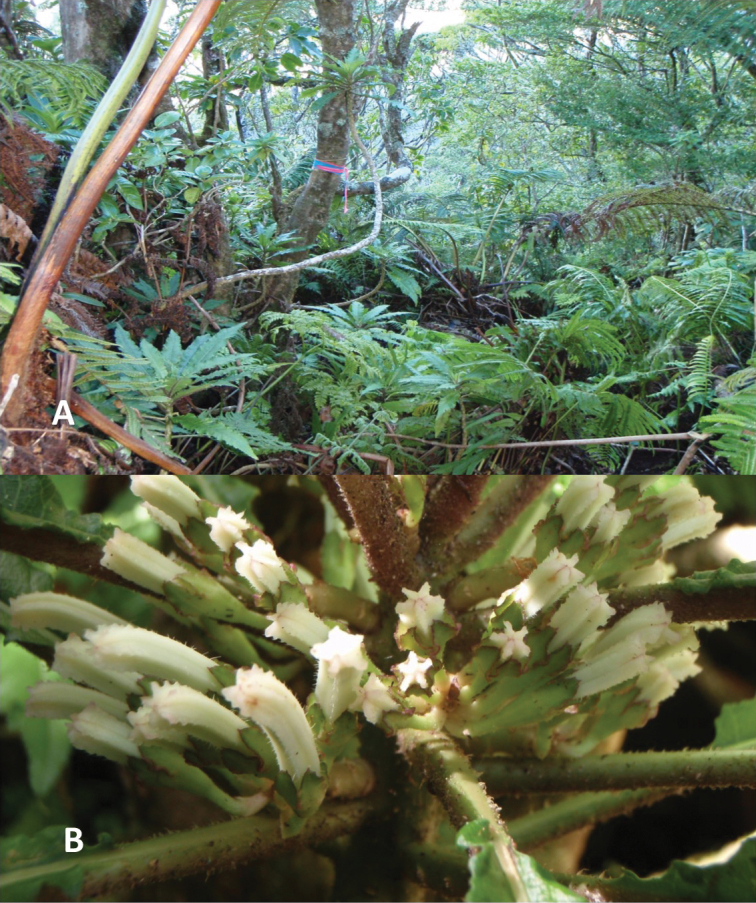
*Cyaneaheluensis***A** habit **B** close up of flower buds prior to anthesis. Note murications on petioles and corolla. Photos by H. Oppenheimer.

**Figure 2.**
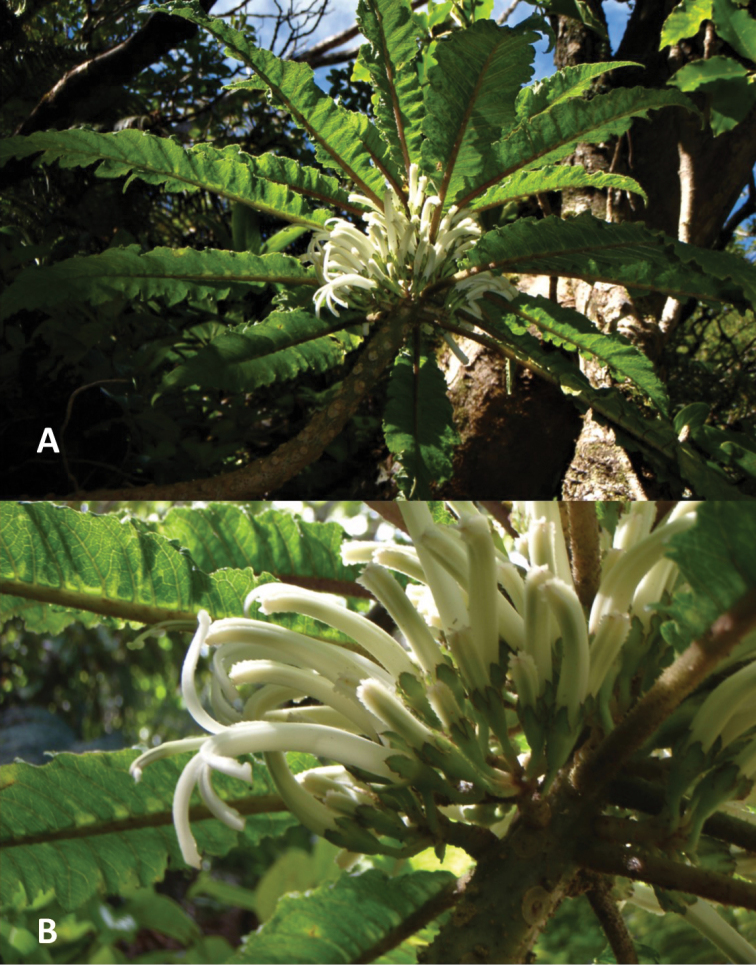
*Cyaneaheluensis***A** flowering stem **B** close-up of flowers. Photos by H. Oppenheimer.

In June of 2010, while near the summit of Helu, Mauna Kahalawai (aka West Maui), the author and Jennifer Higashino spotted through binoculars an unusual *Cyanea*. Upon carefully negotiating down the steep, slippery slope and arriving at the plant, it was immediately recognized as distinct from all the other known taxa on Maui by its habit (Fig. 1), and narrow, shallowly lobed and undulate leaf margins. Flower buds were just beginning to emerge in some of the leaf axils (Fig. 1). Return visits to monitor this individual, and to search for others, found the flowers closer to being at full anthesis in September, when the holotype was collected (Fig. 2). Study of this material revealed it to be distinct from all other known species of the genus, herein described.

## Taxonomic treatment

### Cyanea heluensis

Taxon classificationPlantaeSemaeostomeaeCyaneidae

H.Oppenh. sp. nov.

3D603D27-C0DD-5105-AC0D-B7E995CCAB73

urn:lsid:ipni.org:names:77212950-1

#### Diagnosis.

**Figure 3.**
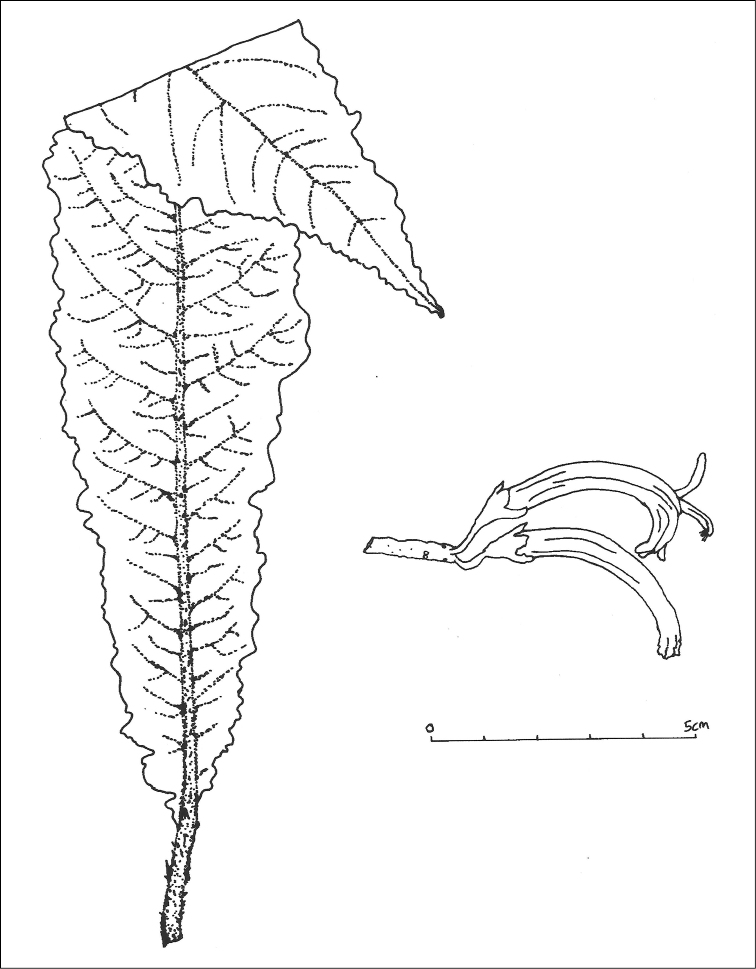
*Cyaneaheluensis*. Leaf (left); flowers (right). Illustration by Anna Palomino. Drawn from H. Oppenheimer et al. #H91007 (BISH) and field images taken by H. Oppenheimer.

Species allied to *C.asplenifolia* (H. Mann) Hillebr., but differs in its very shallowly lobed leaves (vs. deeply lobed to pinnate-pinnatifid), longer and wider corolla, and larger, undulate sepals (Fig. 3).

#### Type.

**Figure 4.**
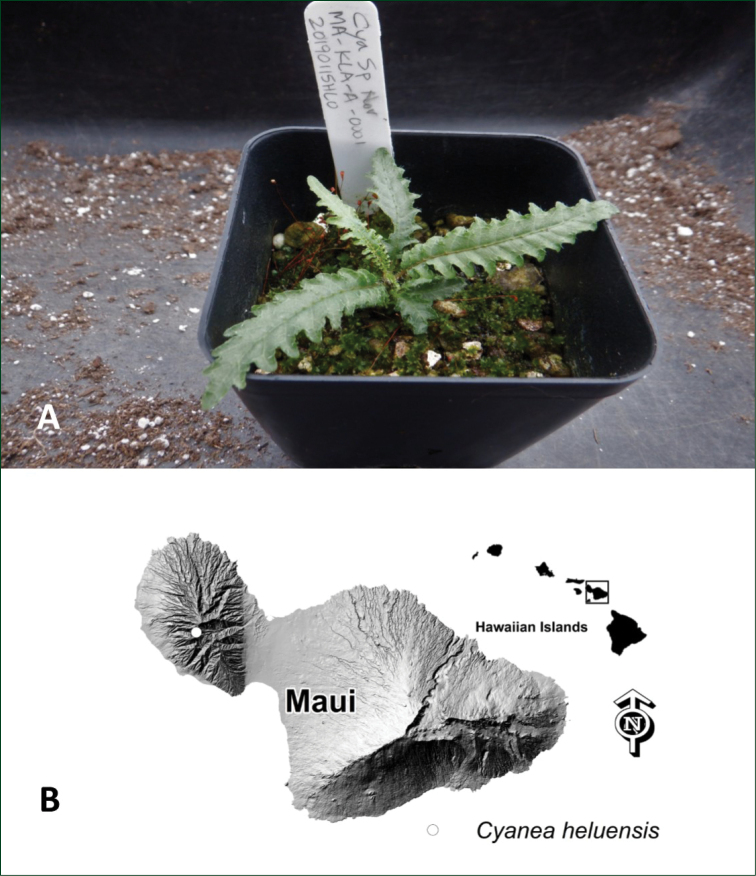
*Cyaneaheluensis***A** seedling at Olinda Rare Plant Facility. Plant is approximately 1 year old **B** distribution *Cyaneaheluensis* of on Maui, Hawai`i. Photo by H. Oppenheimer.

USA, Hawaiian Islands, west Maui, Lahaina District, slopes of Helu, north side, 4160 ft. (1268 m), *H. Oppenheimer, S. Perlman & J.Q.C. Lau #H91007*, 8 Sep 2010 (BISH) (Fig. 4).

#### Description.

Many-branched, sprawling, decumbent to ascending shrub to 3 m long, stems leaning or tangled in adjacent vegetation, occasionally rooting when in contact with wet soil, very sparingly muricate on the lower trunk, denser on younger stems, leaf scars prominent, nearly orbicular, latex white. Leaves clustered at the ends of the branches, petiolate, chartaceous, dark green above, paler on abaxial surface, midrib usually purple on abaxial surface (live material), blade elliptic to oblanceolate, 26–29 cm long x 4–6 cm wide, apex acuminate, base narrowly cuneate, upper surface mostly glabrous, abaxial surface sparsely pubescent, more so on midrib, margins shallowly and irregularly lobed, the lobes 2–6 mm deep, crenate, undulate, petioles 3–4 cm long, sparsely muricate, pubescent. *Juvenile leaves* weakly (Fig. 4) dimorphic, the lobes more distinct than in adult leaves but cut less than ½ way to midrib, leaf apex rounded. *Inflorescence* axillary, among the lower leaves, peduncles 6–15 mm long, 5–10 flowered, glabrous to sparsely pubescent, bracts caducous (not seen), bracteoles caducous, 1 mm, lanceolate to elliptic, acute, sparsely pubescent. *Pedicels* pubescent, 5–9 mm long, bracteoles usually 1–2 per pedicel, persistent, sparsely pubescent, occurring on basal ¼–1/2 of pedicel. *Calyx* green, the lobes deltate, 4–6 mm long x 2–3 mm wide, margins crenate, undulate, glabrous to sparsely pubescent, purple (most apparent in live material), apex acute to short acuminate. *Corolla* white, muricate on corolla lobes especially in bud and distally prior to anthesis, gently curved, 45–55 mm long x 4–5 mm wide, longitudinally pubescent along corolla tube, lobes approximately 1/3 the length of the tube, reflexed, hypanthium 4–5 mm long x 4–5 mm wide, sparsely pubescent. *Berries* small, 5–10 mm, globose, sepals caducous, orange at maturity; *seeds* brown, smooth, round to ellipsoid, < 1 mm.

#### Specimens examined.

USA, Hawaiian Islands. West Maui, Lahaina District, slopes of Helu, south, upper slope of Kaua`ula Valley, *H. Oppenheimer & J. Higashino #H61004*, 3 Jun 2010 (BISH); loc. cit. 18 Oct 2018, *H. Oppenheimer #H101814* (PTBG).

#### Affinities.

Several attempts have been made to divide *Cyanea* into sections (Hillebrand 1888; Rock 1919; Wimmer 1943; Lammers 1990), but none successfully arranges the genus into clear-cut phylogenetic entities. Givnish et al. (1995) divides the genus into two distinct clades: one with purple fruits and another with orange fruits. *Cyaneaheluensis* belongs to a lineage that includes *C.asplenifolia* (H. Mann) Hillebr., its apparent nearest congener and also endemic to Maui, based on their narrow, gently curved tubular, white corollas with murications, and leaf margins that are shallowly lobed to pinnately divided. In contrast to *C.asplenifolia* with its pinnately divided leaves, *C.heluensis* has narrower leaves very shallowly lobed, longer and wider corollas, and larger, undulate sepals. Although Lammers (1990 p. 445) states the berries of *C.asplenifolia* are unknown, this species is now known to have bright orange fruits (pers. obs.). A preliminary phylogenomic analysis by Steve Hunter (Univ. Wisconsin, Madison pers. comm.) using whole chloroplast genome sequences (not nuclear sequences) supports *C.heluensis* as being sister to the small clade formed by *C.asplenifolia* and *C.duvalliorum* Lammers & H. Oppenh.

Based partly on Lammers (1990, 2004) and Givnish et al. (1995), the following key will distinguish *Cyaneaheluensis* from its congeners on Mauna Kahalawai (West Maui).

**Table:** 

1	Fruit purple	**2**
–	Fruit orange	**4**
2(1)	Corolla pubescent	***C.obtusa***
–	Corolla glabrous	**3**
3(2)	Inflorescence 6–25 flowered; peduncles 15–240 mm long	***C angustifolia***
–	Inflorescence 6–14 flowered; peduncles 10–50 mm long	***C.elliptica***
4(1)	All 5 anthers with apical tufts of white hairs	**5**
–	Ventral 2 anthers with apical tufts of white hairs	**7**
5(4)	Leaves lobed	**C.lobatasubsp.lobata**
–	Leaves pinnately divided	**6**
6(5)	Hypanthium campanulate; dorsal anthers 15–16 mm long	***C.mauiensis***
–	Hypanthium obconic; dorsal anthers 9.5–12 mm long	***C.magnicalyx***
7(4)	Corolla laterally compressed	***C.scabra***
–	Corolla tubular, round in cross-section	**8**
8(7)	Corolla blackish purple externally, 60–80 mm long, 6–11 mm wide	***C.macrostegia***
–	Corolla white or white striped with lilac longitudinal lines, 35–55 mm long, 3–5 mm wide	**9**
9(8)	Corolla externally glabrous	***C.kauaulaensis***
–	Corolla externally pubescent, sometimes only on longitudinal lines	**10**
10(9)	Plants unbranched or sparingly branched from base, 0.5–1.5 m tall; corolla pubescent, without murications	***C.kunthiana***
–	Plants branched above base, 1.5–3 m tall; corolla pubescent along longitudinal lines, muricate	**11**
11(10)	Leaves pinnately divided, cut ¾ to ⅞ the distance to the midrib	***C.asplenifolia***
–	Leaves shallowly lobed, cut less than ¼ the distance to the midrib	***C.heluensis***

#### Phenology.

*Cyaneaheluensis* has been observed beginning to flower from mid-summer through October, followed by immature, green fruit observed October to December, maturing in early January.

#### Etymology.

The specific name honors Helu, a peak on Mauna Kahalawai (aka West Maui Mountains) *Lit.* scratch or count (Pukui et al. 1966); + Latin suffix -*ensis*, indicating a place of origin or growth.

#### Habitat & ecology.

*Cyaneaheluensis* occurs in *Metrosideros* Banks ex Gaertn. Lowland Wet Forest (Wagner et al. 1990). The common associated woody elements are species of *Cheirodendron* Nutt. ex Seem., *Clermontia* Gaud., *Coprosma* J.R. Forst. & G. Forst., *Cyrtandra* J.R. Forst. & G. Forst., *Dubautia* Gaud., *Hydrangea* L., *Ilex* L., *Kadua* Cham. & Schltdl., *Myrsine* L., *Perrottetia* Kunth, *Pipturus* Wedd., *Psychotria* L., and *Urera* Gaud. These taxa form a nearly closed canopy with a well-developed understory. Ferns such as species of *Asplenium* L., *Cibotium* Kaulf., *Cyclosorus* Link, *Deparia* Hook. & Grev., *Diplazium* Sw., *Dryopteris* Adans., *Elaphoglossum* Schott ex J. Sm., *Microlepia* C. Presl, *Pteris* L., *Sadleria* Kaulf., *Sticherus* C. Presl, *Tectaria* Cav., and *Vandenboschia* Copel. are prevalent and form a dense ground cover. *Freycinetiaarborea* Gaud. is a widespread liana, and several herbaceous species of *Peperomia* Ruiz & Pav. are also present. The sedges *Machaerinaangustifolia* (Gaudich.) T. Koyama and *Rhynchosporasclerioides* Hook. & Arnott are also frequent. Herbaceous *Hillebrandiasandwicensis* Oliv. and *Gunnerapetaloidea* Gaudich. are distinctive elements of this plant community.

*Cyaneamacrostegia* Hillebr. has been observed infrequently in the general area. *Cyaneaelliptica* (Rock) Lammers, *C.kauaulaensis* H. Oppenh. & Lorence, and *C.scabra* Hillebr. occur to the north, along Kaua`ula Stream. These species are nearly 1500 m away and 500 m or more lower in elevation than *C.heluensis* which is located near the ridgetop. Based on genetic analysis and morphology none of these other taxa appear to be potential hybrid parents. The known populations on Mauna Kahalawai of *Cyaneaasplenifolia*, one of its two closest relatives, are 3,700 m and over 10,000 m away. Populations on Haleakala, East Maui in Makawao Forest Reserve and Haleakala National Park are 40 kilometers and 70 kilometers to the east. Extant populations of *Cyaneaduvalliorum* Lammers & H. Oppenh., the other member of this clade, occur nearly 50 kilometers away on Haleakala. Since these species are also known to be ornithophilus, and populations and densities of native birds have dramatically declined, it is unlikely *C.heluensis* is of hybrid origin.

Soil is of typical basaltic origin derived from the original shied-building Wailuku Volcanic Series (Stearns and MacDonald 1942) with average annual rainfall approximately 3000 mm. (Giambelluca et al. 1986).

The only known plant occurs in deep shade on the upper rim of the steep south side of the large amphitheater-headed Kaua`ula Valley, with a windward aspect. Recently, several other previously undescribed taxa have been discovered in the area, including *Cyaneakauaulaensis* H. Oppenheimer & Lorence (Oppenheimer and Lorence 2012), *Hibiscadelphusstellatus* H. Oppenheimer, Bustamente & Perlman (Malvaceae) (Oppenheimer et. al. 2014), and *Stenogynekauaulaensis* K.R. Wood & H. Oppenheimer (Lamiaceae) (Wood and Oppenheimer 2008). Kaua`ula Valley is an important site not only botanically, but economically (as a water source) as well as culturally and spiritually for Native Hawaiians.

#### Conservation status.

*Cyaneakauaulaensis* should be considered Critically Endangered due to its limited range, low population numbers, lack of population structure with no seedling recruitment, probable loss and decline of most or all of its avian pollinators and dispersal agents, threats such as landslides and treefall, herbivory by alien slugs and rats, and competition with alien plants such as *Ageratinaadenophora* (Sprengel) R.M. King & H. Robinson, *Buddleiaasiatica* Loureiro, *Erigeronkarvinskianus* DC, *Melinisminutiflora* P. Beauv., *Rubusrosifolius* Smith, and *Tibouchinaherbacea* (DC) Cogn. When evaluated using the World Conservation Union (IUCN) criteria for endangerment (IUCN 2001), *Cyaneaheluensis* easily falls into the Critically Endangered (CR) category, which designates species facing a very high risk of extinction in the wild. The CR designation is met when any of the criteria A to E are met. Both Criterion B1 (Extent of Occupancy or EOO) and B2 (Area of Occupancy or AOO) are met with an EOO of less than 100 km^2^ and an AOO of less than 10 km^2^. Criterion D, population size estimated to be fewer than 50 wild individuals is easily met since there is only a single known individual. Criteria A & C address decline in population for which there is no data, although with only a single individual it could reasonably be inferred that this species is in decline. No quantitative analysis predicting the likelihood of extinction (Criterion E) was conducted. The alphanumeric formula CR B1a(I,ii,iv,v)+B2a,b(i,ii,iv,v) represents the current status under IUCN guidelines. Furthermore, *Cyaneaheluensis* should be considered by the US Fish & Wildlife Service as a Candidate for listing as Endangered under the Endangered Species Act of 1973, and a Recovery Plan written, funded, and implemented.

#### Conservation efforts.

Despite several attempts to locate other populations or individuals elsewhere on Helu and adjacent Kaua`ula and Launiupoko Valleys, including the use of ropes and technical gear, only one single plant has ever been observed. Efforts were made shortly after its discovery to collect mature fruit, including covering flowers with protective nylon mesh bags. These efforts failed due to the predation of the exposed peduncles by non-native slugs (e.g. *Deroceruslaevis*). A few short lower branches were collected but only a single one was successfully rooted at the Olinda Rare Plant Facility on Maui, but later died. In 2013, the health and vigor of the plant had declined significantly, but in 2016 it was recovering with new growth and a few new shoots initiated along the main stems. Poor weather prevented helicopter access in late 2017 and early 2018 in attempts to obtain mature fruit. In July of 2018, the plant was showing signs of increased vigor with three ramettes beginning to flower, and several smaller side shoots. Trapping for rats is ongoing, likewise the manual control of weeds. The last flowers of the season and very immature fruit were observed in October of 2018 and October of 2019. A hormone paste was successfully applied in 2018 to the stems to induce branching; three lateral shoots were collected in October of 2019 and sent to the Olinda Rare Plant Facility and Lyon Arboretum Micropropagation Lab on O`ahu. One of these has successfully rooted at Olinda Rare Plant Facility, but the Lyon Arboretum material failed (C. Yamamoto pers. comm.). In January of 2019, a single mature fruit was collected, so resolving which of Givnish’s two clades this new species belongs to. Only a single seed germinated, but it is healthy and continues to grow at the Olinda Rare Plant Facility (Fig. 4) along with the rooted cutting. The Maui Invasive Species Committee (MISC) has been working to control the *Cortaderiajubata* (Lem.) Stapf infestation in adjacent Kaua`ula Valley, and on the surrounding vertical cliffs. The region has for the most part escaped the ravages of introduced feral ungulates due to the extremely rugged topography. However, there has been a small incursion of feral goats and feral pigs approximately 1.5 km to the west and northwest at lower elevation. The Mauna Kahalawai Watershed Partnership (formerly West Maui Mountains Watershed Partnership) has been working to mitigate this incipient yet potentially severe threat. This new species is a target of the Plant Extinction Prevention Program (PEPP), easily meeting the threshold of 50 wild individuals or less for inclusion. The Program strives to collect seeds or cuttings from every individual plant on the list, with propagation of nursery stock, restoration outplantings into appropriate habitat, and *ex situ* seed storage and living collections being the main goals.

## Supplementary Material

XML Treatment for Cyanea heluensis
